# Supplementary material for: Simultaneous Monitoring of Mutation and Chimerism Using Next-Generation Sequencing in Myelodysplastic Syndrome
Source: J Clin Med. 2019 Nov 28;8(12):2077. doi: 10.3390/jcm8122077 (PMC6947461; doi:10.3390/jcm8122077)
Supplement: Supplementary file 1 [file jcm-08-02077-s001.pdf]

Table S1. List of target genes of the NGS panel.

Target genes	Interval	Regions	Size	Coverage
ABCA12	chr2:215797358-216002931	54	7929	100.000
ABL1	chr9:133589707-133761070	12	3529	100.000
ASXL1	chr20:30946579-31025141	17	4720	99.873
ATM	chr11:108098352-108236235	62	9171	100.000
ATRX	chrX:76763829-77041487	36	7543	100.000
ATXN7L1	chr7:105248299-105517004	15	3132	100.000
BCOR	chrX:39909169-39937182	15	5348	100.000
BRAF	chr7:140426294-140624503	21	2379	100.000
BRCC3	chrX:154299803-154348425	11	1021	92.654
CALR	chr19:13049494-13054795	10	1294	100.000
CBL	chr11:119077128-119170491	16	2721	100.000
CBLB	chr3:105377814-105588232	20	3079	100.000
CD101	chr1:117544440-117576723	9	3066	100.000
CEBPA	chr19:33792244-33793425	1	1182	100.000
CREBBP	chr16:3777719-3929917	31	7368	98.846
CSF1R	chr5:149433632-149465990	22	3003	95.771
CSF3R	chr1:36931697-36945097	16	2698	100.000
CTCF	chr16:67644736-67671775	10	2184	100.000
CUX1	chr7:101459311-101926382	34	5433	100.000
DNMT1	chr19:10244343-10311559	43	5292	96.977
DNMT3A	chr2:25457148-25536853	25	2888	99.169
EGFR	chr7:55086971-55273310	31	4084	100.000
EP300	chr22:41489009-41574960	31	7245	100.000
ERG	chr21:39739557-39947624	12	1764	99.206
ETV6	chr12:11803062-12044535	10	1443	100.000
EZH2	chr7:148504738-148544390	21	2456	100.000
FBXW7	chr4:153244033-153332955	14	2618	100.000
FLT3	chr13:28578189-28674647	25	3004	100.000
GATA1	chrX:48649517-48652675	5	1346	100.000
GATA2	chr3:128199862-128205874	5	1443	100.000
GNAS	chr20:57415162-57485884	17	4096	100.000
HIPK2	chr7:139257673-139477422	16	3708	100.000
IDH1	chr2:209101803-209116275	8	1248	100.000
IDH2	chr15:90627498-90645622	11	1359	100.000
INVS	chr9:102866804-103062956	17	3395	99.028
IRF1	chr5:131819643-131825170	9	978	100.000
JAK2	chr9:5021988-5126791	23	3399	100.000
KDM2B	chr12:121867919-122018816	29	4276	100.000
KDM6A	chrX:44732798-44970656	31	4470	100.000
KIT	chr4:55524182-55604723	21	2931	100.000
KMT2A	chr11:118307228-118392887	38	12082	99.479
KMT2D	chr12:49415563-49449107	55	16662	99.424
KRAS	chr12:25362729-25398318	6	708	100.000
LAMB4	chr7:107664484-107763609	35	5507	96.459
MECOM	chr3:168802697-169381160	20	3816	97.642
MET	chr7:116335811-116436178	21	4359	99.014
MLL3	chr7:151833917-152132871	62	15030	99.741
MLL5	chr7:104681400-104753780	27	5743	100.000
MN1	chr22:28146903-28196531	2	3963	100.000
MPL	chr1:43803520-43818443	12	1993	100.000
NCOR2	chr12:124809948-124979797	49	7734	99.289
NF1	chr17:29422226-29705949	63	9011	100.000
NLRP1	chr17:5405134-5487277	18	4493	97.930
NOTCH1	chr9:139390523-139440238	34	7668	100.000
NPM1	chr5:170814953-170837569	12	894	100.000
NRAS	chr1:115251156-115258781	4	570	100.000
NRD1	chr1:52254908-52344287	34	3696	99.026

NUP98	chr11:3692612-3803347	35	5567	100.000
OCA2	chr15:28000534-28327020	24	2573	100.000
PDGFRA	chr4:55106220-55161439	24	3450	98.174
PHF12	chr17:27233201-27278622	17	3519	100.000
PHF6	chrX:133511648-133559360	9	1207	100.000
PRPF40B	chr12:50017374-50037975	26	2682	100.000
PRPF8	chr17:1553953-1587865	42	7151	100.000
PTPN11	chr12:112856916-112942568	16	1822	98.024
RAD21	chr8:117859739-117878968	13	1896	100.000
RAD50	chr5:131893017-131978781	27	4211	95.559
RINT1	chr7:105172763-105207758	16	2417	98.428
ROBO1	chr3:78648063-79639061	34	5223	100.000
ROBO2	chr3:75986645-77695209	32	4862	100.000
RUNX1	chr21:36164432-36421196	11	1584	97.790
RUNX1T1	chr8:92972470-93115112	20	2350	98.596
SETBP1	chr18:42281312-42643663	6	4980	100.000
SF3A1	chr22:30730583-30752781	16	2382	100.000
SF3B1	chr2:198257027-198299723	27	4045	100.000
SMC1A	chrX:53407024-53449549	26	3882	100.000
SMC3	chr10:112327575-112364060	29	3654	100.000
SRSF2	chr17:74732243-74733242	2	666	100.000
STAG2	chrX:123156478-123234447	34	3861	100.000
TET1	chr10:70332096-70451571	11	6411	100.000
TET2	chr4:106111627-106197676	10	6165	100.000
TP53	chr17:7565257-7579912	14	1378	91.655
TP53BP1	chr15:43699581-43785241	31	6130	100.000
U2AF1	chr21:44513212-44527604	9	790	94.430
U2AF2	chr19:56166471-56185434	14	1541	100.000
WT1	chr11:32410604-32456891	11	1568	100.000
ZRSR2	chrX:15808619-15841365	12	1690	80.592

Table S2. List of SNPs included in the NGS chimerism calculation.

Marker number	Gene	Chr	Coordinate	dbSNP ID	ExAC (Total)	ExAC (East Asian)	KRGDB	Ref	Alt	Read depth (mean ± SD)	%BE	%ME
1	*CD101*	1	117560818	rs3736908	0.35	0.61	0.52	A	G	1313.08±260.08	0.06	4.04
2	*DNMT3A*	2	25458546	rs2304429	0.52	0.29	0.3	C	T	1215.08±225.42	0.05	2.27
3	*DNMT3A*	2	25463483	rs2289195	0.41	0.24	0.26	G	A	884.92±152.59	0.07	-0.56
4	*DNMT3A*	2	25469913	rs2276599	0.71	0.6	0.57	C	T	766.33±124.52	0.02	-0.7
5	*SF3B1*	2	198257795	rs4685	0.66	0.54	0.64	T	C	2257.00±365.74	0.06	-1.55
6	*SF3B1*	2	198263146	rs16865262	0.2	0.27	0.3	T	C	1795.33±301.50	0.03	-1.72
7	*SF3B1*	2	198265173	rs788017	0.65	0.53	0.62	T	A	1027.83±205.29	0.09	-8.03
8	*SF3B1*	2	198265526	rs788018	0.66	0.53	0.64	A	G	2364.58±368.06	0.10	5.43
9	*SF3B1*	2	198283305	rs788023	0.66	0.53	0.63	T	C	1260.83±210.06	0.06	-3.45
10	*ABCA12*	2	215820013	rs10498027	0.35	0.23	0.21	G	A	2304.00±491.21	0.10	-3.49
11	*ABCA12*	2	215833415	rs4673925	0.51	0.27	0.23	A	G	1163.50±234.53	0.08	4.23
12	*ABCA12*	2	215865575	rs10498030	0.26	0.38	0.36	T	C	2429.33±437.68	0.07	-1.96
13	*ABCA12*	2	215901774	rs17501837	0.21	0.4	0.47	C	T	1044.75±264.25	0.08	6.99
14	*ABCA12*	2	215928972	rs1523721	0.57	0.7	0.74	G	C	1137.17±209.76	0.13	-1.39
15	*ROBO2*	3	77684222	rs3821735	0.08	0.19	0.21	C	T	1030.67±189.65	0.12	-1.25
16	*ROBO1*	3	78700901	rs6795556	0.22	0.21	0.27	G	T	1774.42±344.02	0.07	2.81
17	*ROBO1*	3	78717343	rs2271151	0.23	0.22	0.27	C	T	2217.83±419.49	0.09	3.83
18	*ROBO1*	3	78737962	rs967454	0.44	0.21	0.21	G	A	1191.67±235.73	0.10	-2.14
19	*ROBO1*	3	78796078	rs2304503	0.5	0.23	0.21	C	A	1149.92±257.54	0.09	-8.24
20	*CBLB*	3	105389153	rs11713094	0.22	0.21	0.23	A	G	1382.33±248.81	0.07	4.83
21	*CBLB*	3	105422844	rs2305037	0.73	0.76	0.77	C	T	513.00±130.18	0.07	4.41
22	*CBLB*	3	105438957	rs2305036	0.23	0.24	0.23	T	G	1605.33±268.66	0.04	-9.46
23	*GATA2*	3	128204951	rs2335052	0.2	0.38	0.4	C	T	711.17±128.09	0.11	-0.23
24	*TET2*	4	106155185	rs12498609	0.06	0.2	0.21	C	G	2234.00±416.36	0.07	1.49
25	*TET2*	4	106196092	rs2647243	0.91	0.57	0.59	C	T	1254.33±219.94	0.05	5.35
26	*TET2*	4	106196951	rs2454206	0.3	0.21	0.21	A	G	2294.33±458.37	0.07	6.13
27	*FBXW7*	4	153252061	rs10033601	0.38	0.45	0.44	A	G	1591.25±278.16	0.09	3.33
28	*IRF1*	5	131819798	rs2070731	0.33	0.34	0.24	T	C	853.92±177.38	0.05	-3.02
29	*IRF1*	5	131819800	rs2070730	0.35	0.34	0.24	G	A	841.67±173.36	0.04	-3.56
30	*IRF1*	5	131822055	rs9282762	0.35	0.34	0.24	T	C	1099.58±204.94	0.02	-9.04
31	*IRF1*	5	131822224	rs9282761	0.35	0.34	0.24	T	C	1051.50±188.73	0.03	-4.92
32	*CSF1R*	5	149435759	rs216136	0.53	0.47	0.39	G	A	1316.67±229.41	0.15	0.06
33	*CSF1R*	5	149456811	rs3829987	0.1	0.22	0.23	G	A	1243.67±227.56	0.17	-6.09
34	*CSF1R*	5	149457678	rs2228422	0.51	0.22	0.21	G	A	1600.33±300.18	0.10	-5.97
35	*NPM1*	5	170819887	rs3830035	0.41	0.55	0.6	G	A	1998.50±367.12	0.05	-1.06
36	*NPM1*	5	170820024	rs3830036	0.41	0.54	0.6	G	A	1753.67±312.97	0.08	-3.67
37	*EGFR*	7	55214348	rs2072454	0.51	0.37	0.37	C	T	1514.50±282.44	0.06	-0.77
38	*EGFR*	7	55221655	rs4947986	0.31	0.64	0.65	G	A	984.50±166.23	0.17	0.83
39	*EGFR*	7	55229255	rs2227983	0.29	0.53	0.57	G	A	961.00±158.68	0.09	-3.18
40	*EGFR*	7	55238874	rs2227984	0.38	0.54	0.61	T	A	1784.17±354.45	0.04	-2.37
41	*EGFR*	7	55268916	rs2293347	0.14	0.27	0.34	C	T	2004.42±399.01	0.06	1.87
42	*KMT2E*	7	104717517	rs2240455	0.22	0.64	0.68	C	T	1901.33±392.28	0.04	6.93
43	*KMT2E*	7	104741842	rs10953468	0.32	0.29	0.28	A	T	961.33±231.90	0.08	1.83
44	*KMT2E*	7	104742054	rs11976329	0.21	0.57	0.63	C	T	1214.83±295.25	0.06	4.87
45	*LAMB4*	7	107745029	rs2074748	0.05	0.16	0.21	A	G	2437.83±455.63	0.09	3.16

46	*LAMB4*	7	107746253	rs420753	0.39	0.38	0.41	A	G	1296.92±277.39	0.05	4.28
47	*EZH2*	7	148508833	rs2072407	0.67	0.67	0.65	A	G	1189.67±222.65	0.14	2.86
48	*EZH2*	7	148543525	rs10274535	0.7	0.66	0.65	A	G	1588.33±307.23	0.11	-0.06
49	*KMT2C*	7	151842397	rs2240819	0.07	0.17	0.22	C	T	1853.25±346.13	0.09	3.73
50	*KMT2C*	7	151859683	rs74483926	0.05	0.18	0.22	G	A	2247.17±411.58	0.10	1.71
51	*KMT2C*	7	151873853	rs6464211	0.24	0.32	0.32	C	T	2511.17±449.18	0.07	2.49
52	*KMT2C*	7	151874498	rs10252263	0.07	0.17	0.22	C	T	2391.17±405.30	0.11	1.76
53	*JAK2*	9	5050706	rs2230722	0.32	0.27	0.24	C	T	2071.67±417.57	0.09	5.97
54	*JAK2*	9	5090934	rs2274649	0.28	0.26	0.23	A	T	682.58±149.04	0.02	5.01
55	*ABL1*	9	133753980	rs34001282	0.01	0.02	0.04	C	G	918.25±157.40	0.19	9.18
56	*ABL1*	9	133760635	rs35445683	0.34	0.03	0.03	A	G	1261.83±230.68	0.08	4.17
57	*ABL1*	9	133761001	rs1056171	0.55	0.6	0.6	A	G	1168.17±230.30	0.11	5.94
58	*NOTCH1*	9	139407932	rs2229971	0.4	0.79	0.78	A	G	1164.17±189.92	0.17	2.63
59	*TET1*	10	70332580	rs10823229	0.33	0.38	0.35	A	G	2344.67±408.04	0.09	2.9
60	*TET1*	10	70332672	rs12773594	0.18	0.17	0.22	T	A	2127.42±365.70	0.08	-3.24
61	*TET1*	10	70332862	rs12221107	0.12	0.15	0.2	C	T	1972.58±380.83	0.06	4.25
62	*SMC3*	10	112343591	rs11195199	0.15	0.25	0.31	G	A	1198.08±317.27	0.10	-2.09
63	*NUP98*	11	3714489	rs1875	0.21	0.31	0.28	C	T	1935.92±355.23	0.08	0.99
64	*WT1*	11	32410774	rs1799937	0.35	0.72	0.69	A	G	1138.67±217.44	0.12	9.49
65	*WT1*	11	32417945	rs16754	0.24	0.69	0.67	T	C	1611.00±285.09	0.09	-1.59
66	*ATM*	11	108225483	rs664982	0.53	0.41	0.46	C	T	1166.42±274.58	0.05	2.42
67	*ETV6*	12	12006544	rs17210957	0.18	0.4	0.43	G	A	1251.92±256.22	0.10	-0.64
68	*KMT2D*	12	49424534	rs11168830	0.1	0.26	0.29	G	A	726.25±153.13	0.09	-2.72
69	*KMT2D*	12	49425978	rs3741622	0.32	0.44	0.36	T	C	1092.58±151.95	0.07	-4.38
70	*KDM2B*	12	121881848	rs10849885	0.38	0.44	0.39	G	A	570.17±110.48	0.07	-4.84
71	*NCOR2*	12	124885249	rs2293514	0.66	0.74	0.71	C	T	500.17±100.12	0.09	8.59
72	*NCOR2*	12	124968349	rs747443	0.64	0.55	0.54	T	C	574.75±108.73	0.09	-3.23
73	*FLT3*	13	28589267	rs4073630	0.48	0.41	0.38	C	T	1280.17±222.92	0.09	4.83
74	*FLT3*	13	28592546	rs17086226	0.24	0.25	0.27	T	C	1389.58±254.30	0.06	-2.01
75	*FLT3*	13	28607989	rs2491223	0.71	0.75	0.7	T	G	1219.08±204.80	0.12	-2.66
76	*FLT3*	13	28609825	rs2491227	0.7	0.75	0.7	A	G	1400.58±252.82	0.14	1.45
77	*FLT3*	13	28610183	rs2491231	0.7	0.75	0.7	A	G	1396.00±234.52	0.13	-1.5
78	*FLT3*	13	28624294	rs1933437	0.61	0.77	0.76	G	A	2277.25±418.11	0.07	-2.23
79	*OCA2*	15	28197037	rs1800414	0.04	0.6	0.54	T	C	1714.08±363.04	0.15	-5.86
80	*OCA2*	15	28235773	rs1800404	0.64	0.38	0.44	C	T	1040.75±189.17	0.13	-1.67
81	*TP53BP1*	15	43720197	rs550239	0.19	0.41	0.37	C	T	1214.58±218.31	0.09	0.52
82	*TP53BP1*	15	43724646	rs2602141	0.36	0.41	0.45	T	G	2499.42±447.23	0.04	-1.76
83	*TP53BP1*	15	43748304	rs690367	0.36	0.41	0.45	A	G	2571.17±419.73	0.07	3.05
84	*TP53BP1*	15	43762196	rs689647	0.2	0.41	0.45	C	T	1570.42±262.14	0.05	-0.21
85	*TP53BP1*	15	43767774	rs560191	0.36	0.41	0.45	G	C	1289.58±262.24	0.09	-1.84
86	*TP53BP1*	15	43784475	rs2439831	0.19	0.41	0.45	C	T	1910.67±345.46	0.04	3.39
87	*IDH2*	15	90627616	rs60147683	0.26	0.23	0.25	T	C	694.92±140.87	0.07	-3.08
88	*NLRP1*	17	5425077	rs11651270	0.45	0.24	0.25	T	C	846.50±147.64	0.03	3.94
89	*NLRP1*	17	5487164	rs884367	0.25	0.67	0.64	C	G	773.92±146.88	0.10	7.3
90	*TP53*	17	7579801	rs1642785	0.67	0.57	0.57	G	C	612.42±137.84	0.10	2.77
91	*NF1*	17	29486152	rs2952976	0.63	0.56	0.52	G	A	1260.00±240.34	0.08	0.94
92	*NF1*	17	29508775	rs1801052	0.62	0.56	0.53	G	A	1133.50±204.72	0.11	-5.64
93	*NF1*	17	29541437	rs2905876	0.61	0.56	0.53	T	C	911.33±171.70	0.04	-6.44

94	*NF1*	17	29553485	rs2285892	0.38	0.52	0.59	G	A	1689.25±317.33	0.13	-0.75
95	*NF1*	17	29653293	rs9894648	0.6	0.44	0.46	T	C	1299.08±245.00	0.10	0.38
96	*NF1*	17	29679246	rs964288	0.53	0.35	0.34	G	A	956.58±175.84	0.19	-0.34
97	*SETBP1*	18	42456653	rs663651	0.52	0.5	0.47	G	A	1344.58±250.66	0.05	-8.2
98	*SETBP1*	18	42532606	rs3744825	0.12	0.28	0.3	G	A	2366.25±437.99	0.07	-3.19
99	*DNMT1*	19	10251747	rs2290684	0.48	0.31	0.36	A	G	1004.17±179.32	0.07	4.26
100	*DNMT1*	19	10265248	rs2114724	0.52	0.7	0.64	T	C	781.33±153.65	0.07	-1.72
101	*DNMT1*	19	10267077	rs2228611	0.52	0.7	0.56	T	C	1347.25±264.78	0.09	-2.88
102	*DNMT1*	19	10271034	rs2241531	0.15	0.47	0.42	C	G	1478.83±256.32	0.13	4.53
103	*DNMT1*	19	10271141	rs11672909	0.14	0.43	0.38	A	C	1289.33±234.49	0.09	3.86
104	*DNMT1*	19	10273372	rs2228612	0.14	0.42	0.34	T	C	995.33±165.05	0.11	-4.41
105	*CALR*	19	13054781	rs1049481	0.62	0.81	0.78	G	T	799.92±146.74	0.03	-0.35
106	*U2AF2*	19	56185250	rs664684	0.74	0.46	0.57	G	A	759.67±125.97	0.06	-0.31
107	*ASXL1*	20	30954295	rs2295454	0.42	0.78	0.71	A	G	1693.83±394.32	0.09	1.1
108	*ASXL1*	20	31019024	rs2295765	0.84	0.81	0.75	C	T	1602.33±418.93	0.04	2.29
109	*ASXL1*	20	31024274	rs4911231	0.42	0.78	0.71	T	C	1624.42±392.76	0.09	-3.7
110	*GNAS*	20	57478807	rs7121	0.54	0.65	0.55	C	T	1911.33±357.11	0.09	1.99
111	*SF3A1*	22	30738161	rs9608885	0.66	0.76	0.79	C	T	1421.17±257.06	0.04	1.81
112	*EP300*	22	41551039	rs20552	0.66	0.81	0.77	T	A	2483.08±441.59	0.09	-2.84
113	*EP300*	22	41568480	rs2076577	0.61	0.59	0.63	T	C	1192.00±203.00	0.07	-1.98
114	*EP300*	22	41569609	rs2076578	0.3	0.47	0.54	C	T	892.17±197.55	0.04	4.73
115	*ZRSR2*	X	15836648	rs5936062	0.53	0.65	0.55	T	G	706.17±197.27	0.09	-3.17
116	*BCOR*	X	39932907	rs6520618	0.25	0.7	0.58	T	C	877.92±247.79	0.09	-5.68
117	*KDM6A*	X	44929077	rs2230018	0.13	0.39	0.38	C	A	1352.92±343.34	0.06	0.33
118	*KDM6A*	X	44938563	rs20539	0.25	0.32	0.37	G	A	1092.42±289.42	0.08	-0.35
119	*SMC1A*	X	53449568	rs1264011	0.52	0.53	0.43	G	A	571.92±157.58	0.10	-1.72
120	*ATRX*	X	76937963	rs3088074	0.38	0.45	0.46	G	C	1812.75±515.93	0.10	-2.3
121	*ATRX*	X	76940534	rs35268552	0.37	0.46	0.46	A	G	846.50±231.84	0.06	4.35

Chr, chromosome; KRGDB, Korean Reference Genome Database; Ref, reference sequence; Alt, alternative sequence; BE, Background error; ME, Measurement error
